# Description of high-altitude, cold-adaptive, metabolically versatile *Dyadobacter aurulentus* sp. nov. isolated from Western Himalayan farmland soils

**DOI:** 10.1128/spectrum.01145-25

**Published:** 2025-10-09

**Authors:** Amit Yadav, Kiran Kirdat, Vipool Thorat, Ngangyola Tuikhar, Kirti Chundawat, Tushar Lodha, Bhavesh Tiwarekar, Umera Patwekar, Malad Mubarak, Shuchi Shastri, Saurabh Kumar, Yogesh Shouche, Reeta Goel

**Affiliations:** 1 National Centre for Microbial Resource, National Centre for Cell Science, University of Pune campus, Pune, India; 2 CSIR-Central Institute of Medicinal and Aromatic Plants, Lucknow, India; 3 Department of Biotechnology and Microbiology, Mohan Lal Sukhadia University 29798 , Udaipur, India; 4 Bioenergy Group, Agharkar Research Institute, Pune, India; 5 Ajinkya DY Patil University, Pune, India; 6 Department of Botany, Fergusson College, Pune, India; 7 Department of Microbiology, College of Basic Sciences and Humanities, G. B. Pant University of Agriculture and Technology 564717, Pantnagar, Uttarakhand, India; 8 Division of Crop Research, ICAR-Research Complex for Eastern Region, Patna, India; 9 Department of Biotechnology, Institute of Applied Sciences & Humanities, GLA University, Mathura, India; University of Delhi, Delhi, India

**Keywords:** *Dyadobacter*, *Spirosomaceae*, high-altitude soil, Western Himalaya

## Abstract

A novel bacterial strain, designated UC10^T^, was isolated from cold, high-altitude farmland soil in the Gangotri region of the Western Himalayas, India. The strain is Gram-stain-negative, aerobic, non-spore forming, and non-motile, forming golden colonies that produce a flexirubin-like pigment. Strain UC10^T^ grows over a broad range of temperatures (5°C–30°C), pH (6–11), and salinities (up to 4% NaCl), with optimal growth at 30°C, pH 7.0, and 1% NaCl. The nearly full-length 16S rRNA gene sequence (MK743979) shares 98.95% similarity with *Dyadobacter luticola*, followed by 97.68% with *Dyadobacter crusticola* and 97.40% with *Dyadobacter koreensis*, and phylogenetic analysis places UC10ᵀ in a distinct clade within the genus *Dyadobacter*. Whole-genome phylogenetic analyses revealed that UC10^T^ is closely related to *Dyadobacter linearis*, *D. crusticola*, and *D. luticola* but is clearly distinguished from them by low average nucleotide identity (<81%), digital DNA–DNA hybridization (<24%), and amino acid identity (<80%) values. The genome of UC10^T^ is 6.93 Mb with a G+C content of 46.5 mol% and encodes multiple cold adaptation-related genes, including cold-shock proteins and fatty acid desaturases. The strain also harbors genes for aromatic compound degradation and demonstrated the ability to grow in minimal medium containing sodium benzoate as the sole carbon source. Additionally, fatty acid and polar lipid profiles of UC10^T^ revealed unique compositions, further supporting its differentiation. The combined genomic, phenotypic, and chemotaxonomic evidence supports the designation of strain UC10^T^ as representing a novel species, for which the name *Dyadobacter aurulentus* sp. nov. is proposed. The type strain is UC10^T^ (= MCC 4019^T^ = KCTC 72455^T^ = JCM 34514^T^).

**IMPORTANCE:**

High-altitude, cold habitats such as the Gangotri region of the Western Himalayas remain underexplored for culturable microbial diversity. Here, we describe *Dyadobacter aurulentus* sp. nov., a novel cold-adapted species isolated from such an environment. This strain demonstrates unique ecological and metabolic traits, including growth at low temperatures and degradation of aromatic compounds like sodium benzoate. Genomic analysis revealed key cold adaptation features such as cold-shock proteins, fatty acid desaturases, nitrate assimilation pathways, and multidrug resistance genes, supporting survival in nutrient-limited, low-temperature soils. The strain’s distinct chemotaxonomic profile, marked by elevated C_16:0_ and unique polar lipids, underscores its ecological specialization. Together, these features point to its potential utility in bioremediation and cold-environment biotechnology. This study broadens our understanding of the adaptive strategies and ecological functions of *Dyadobacter* spp. in extreme environments, with implications for bioprospecting cold-active enzymes and understanding resistance evolution in high-altitude microbial communities.

## INTRODUCTION

The genus *Dyadobacter* comprises Gram-stain-negative, rod-shaped bacteria belonging to the family *Spirosomaceae* within the phylum *Bacteroidota* (1). The genus name *Dyadobacter* is derived from the Greek *dyas* (pair) and *bacter* (rod), referencing the organism’s tendency to form cells in pairs (2). Members of this genus are strictly aerobic and typically produce flexirubin-type pigments, which impart a distinctive orange to yellow colony coloration (3). *Dyadobacter* spp. exhibit broad ecological diversity, having been isolated from terrestrial, aquatic, plant-associated, and cold environments.

Several species have been recovered from soil ecosystems, including *Dyadobacter fermentans* (2), *Dyadobacter soli* (4), and *Dyadobacter alkalitolerans* (5), where they contribute to microbial community structure and nutrient cycling. Other species such as *Dyadobacter arcticus* (6), *Dyadobacter frigoris* (7), and *Dyadobacter psychrophilus* (8) originate from cold or Arctic habitats, reflecting adaptation to psychrotolerant or psychrophilic lifestyles. Species like *Dyadobacter chenhuakuii* (9) and *Dyadobacter endophyticus* (10) have been isolated from plant-associated environments, while *Dyadobacter linearis* and *Dyadobacter helix* (11), both recovered from drinking water systems, represent novel morphologies and ecological settings; notably, *D. helix* exhibits a rare helical cell shape. Taxonomically, the genus was initially placed within the family *Cytophagaceae* but was later reclassified under *Spirosomaceae*. As of August 2025, 30 species are validly described within this genus (3), many contributing to our understanding of microbial diversity in extreme or underexplored environments.

The Western Himalayas are characterized by extreme environmental conditions such as low temperatures, hypoxia, intense UV radiation, and nutrient-poor soils. These high-altitude habitats remain relatively underexplored for culturable microorganisms despite their ecological significance. Microorganisms thriving in such regions often exhibit unique physiological, metabolic, and genomic adaptations for cold survival, including cold-active enzymes, pigment production, flexible membrane lipid compositions, and specialized stress response pathways (12). These features not only support microbial persistence in challenging environments but also make cold-adapted microbes valuable for ecological studies and biotechnological applications. Cold ecosystems, including alpine and polar soils, glacier sediments, and cryo-environments, are known to host taxonomically and metabolically diverse microbial communities, often dominated by members of the phyla *Actinomycetota*, *Bacteroidota*, *Pseudomonadota*, and *Bacillota* (13, 14).

In our previous studies (15, 16), we examined how altitude influences microbial diversity across cold desert soils of the Indian Himalayas. We observed strong correlations between elevation, pH, and nutrient profiles, which were accompanied by shifts in the dominance of bacterial phyla, from *Pseudomonadota* and *Acidobacteriota* at higher altitudes to *Bacteroidota* and *Bacillota* at lower elevations. These studies also led to the isolation of novel psychrotrophic strains such as *Arthrobacter humicola* and several *Pseudomonas* spp., revealing unique microbial adaptations in high-altitude, low-temperature ecosystems.

In the present study, we describe the isolation and polyphasic taxonomic characterization of a novel *Dyadobacter* strain, designated UC10^T^, recovered from high-altitude agricultural soil in the Gangotri region of the Western Himalayas (15). Strain UC10^T^ demonstrated cold-adaptive growth and exhibited distinct phenotypic and chemotaxonomic traits. Comprehensive analysis, including 16S rRNA gene sequencing, whole-genome comparisons, fatty acid and polar lipid profiling, and biochemical testing, revealed that UC10^T^ represents a novel species within the genus *Dyadobacter*. We further explore the ecological significance of this strain, its genomic features related to cold adaptation and aromatic compound degradation, and its potential relevance for environmental or biotechnological applications. Based on these findings, we propose the name *Dyadobacter aurulentus* sp. nov. for this novel taxon.

## MATERIALS AND METHODS

### Isolation and culture conditions

Soil samples were collected in the summer of 2016 from high-altitude agricultural land in Gangotri, Uttarakhand, India (30.9947°N, 78.9398°E; 3,415 m above sea level), as described previously (16). The top 5 cm of surface soil was aseptically sampled using a sterile auger, homogenized, and transferred into sterile polyethylene bags. Samples were transported to the laboratory under cool conditions (4°C–8°C) to preserve microbial viability.

Cold-adapted bacteria were isolated using an *in situ* diffusion chamber technique (15, 16). Briefly, soil was inoculated into sealed diffusion chambers, which were incubated aerobically for 15 days at 12°C ± 2°C within a moist soil bed prepared from the same sampling site. Following incubation, emerging colonies were subcultured on nutrient agar (M001; HiMedia, India) and Burk’s medium (M707, HiMedia). The strain UC10^T^, a focal isolate of this study, was identified through 16S rRNA gene sequencing (16).

Pure cultures were preserved at –80°C in 1× phosphate-buffered saline (M1866, HiMedia) containing 20% glycerol (vol/vol) (MB060, HiMedia). Strain UC10^T^ was revived by streaking onto nutrient agar plates and incubating at 5°C, 10°C, 15°C, 20°C, 25°C, and 30°C for up to 4 days. Colonies with characteristic golden pigmentation were selected for further analysis.

The type strain UC10^T^ was deposited in the Microbial Culture Collection (MCC) at the National Centre for Cell Science, India; the Korean Collection for Type Cultures (KCTC), South Korea; and the Japan Collection of Microorganisms (JCM), Japan.

### Identification and 16S rRNA gene phylogeny

Genomic DNA from strain UC10^T^ was extracted using the cetyltrimethylammonium bromide method following standard protocols (17). DNA quality and quantity were assessed using both a UV spectrophotometer (NanoDrop One; Thermo Fisher Scientific, USA) and a Qubit fluorometer (Thermo Fisher Scientific). The nearly full-length 16S rRNA gene was amplified by PCR using the universal bacterial primers 27F and 1492R (18). PCR products were purified and sequenced using the same primer set (19). The resulting sequence was then deposited in GenBank.

The 16S rRNA gene sequence was compared against reference sequences in the EzBioCloud database (20) for initial taxonomic identification and phylogenetic affiliation. Phylogenetic trees were reconstructed using the neighbor-joining, maximum-likelihood, and maximum-parsimony methods implemented in MEGA (version 11) (21). Appropriate evolutionary models were selected based on the best-fit model criterion for each method to ensure reliable tree topologies. Tree robustness was assessed through bootstrap analysis based on 1,000 replicates.

### Genome sequencing and analysis

The genome of strain UC10^T^ (MCC 4019) was sequenced using a hybrid approach combining Illumina HiSeq (150 × 2 paired-end chemistry) and Oxford Nanopore Technologies (ONT) platforms. Quality control of raw sequencing reads was performed using FastQC (version 0.11.9) (22) to assess base quality scores and detect overrepresented sequences. Adapter trimming and quality filtering of Illumina reads were carried out using Fastp (version 0.20.0) (23). ONT reads were base-called using Guppy (with a Q7 quality threshold), and reads shorter than 1 kb were filtered out using NanoFilt (version 2.8.0) (24).

A hybrid genome assembly was generated using SPAdes (version 3.11.1) (25), integrating high-quality Illumina and ONT data. The quality of the assembled genome was evaluated using QUAST (version 4.6.3) (26), which provided key metrics such as total length, N50, and number of contigs. Genome completeness and contamination were assessed with CheckM (version 1.1.3) (27).

Genomic distinctiveness was assessed based on DNA G+C content and whole-genome relatedness metrics. Average nucleotide identity (ANI) was calculated using OrthoANI (28), while digital DNA–DNA hybridization (dDDH) was estimated using the Genome-to-Genome Distance Calculator (GGDC) (version 3.0), applying formula 2 (29). Average amino acid identity (AAI) was computed using both the EzAAI pipeline (30) and the AAI calculator (31).

Phylogenomic analysis was performed using BPGA (version 1.3.0) (32) and UBCG (version 3.0) (33). BPGA employed USEARCH (34) to identify orthologous protein clusters, which were aligned and concatenated to construct a neighbor-joining tree using MEGA (version 11) (21) based on 26,112 aligned amino acid positions. UBCG was used to extract and align core genes from UC10^T^ and its closest relatives to infer phylogenomic relationships.

Genome annotation was performed using the NCBI Prokaryotic Genome Annotation Pipeline (version 6.6) (35), which identified coding sequences, rRNAs, tRNAs, and other genomic features. Functional annotation was further refined using eggNOG-mapper (version 2) (36) to assign orthologous groups and InterProScan (37) to detect conserved domains and motifs. Kyoto Encyclopedia of Genes and Genomes (KEGG) Orthology identifiers were assigned via BlastKOALA (38) for metabolic pathway reconstruction.

Comparative genomic insights were visualized using a Venn diagram (39) to highlight shared and unique Clusters of Orthologous Groups (COGs). Functional category distributions were derived using DeepNOG (40), excluding predictions with confidence scores below 0.5. The distribution of functional COG categories across UC10^T^ and related *Dyadobacter* spp. was illustrated using bar plots based on gene counts per category.

For comparative polyphasic characterization, the type strains *Dyadobacter luticola* T17^T^ (KCTC 52981^T^) (41), *D. linearis* AB67^T^ (LMG 32342^T^) (11), and *Dyadobacter crusticola* CP183-8^T^ (DSM 16708^T^) (42) were obtained from their respective culture collections.

### Morphological, physiological, and biochemical characteristics

Morphological features, including cell shape and size of strain UC10^T^, were examined using scanning electron microscopy (SEM; Zeiss EVO 18, version 6.02). A freshly grown colony was suspended in sterile phosphate-buffered saline, centrifuged, and fixed in 2.5% glutaraldehyde (Merck, Germany) at 4°C for 24 hours. The fixed pellet was dehydrated through a graded ethanol series (20%, 35%, 50%, 70%, 90%, and twice with 100%) for 10 minutes at each step (43), sputter-coated with gold particles (Model SC7620; Quorum Technologies, UK), and visualized under SEM.

The Gram reaction was determined using a commercial Gram stain kit (K001-KT, Himedia) and observed under a light microscope (BX53, Olympus, USA). Motility was assessed using the hanging drop technique and confirmed on semisolid nutrient agar supplemented with triphenyl-tetrazolium chloride (65599; SRL Diagnostics, India), following the method described in reference 44. Oxidase activity was tested using oxidase disks (DD018, Himedia), with development of a purple coloration indicating a positive reaction. Catalase activity was evaluated by observing effervescence after the addition of 3% (vol/vol) hydrogen peroxide (PCT1511, Himedia).

Growth was tested on a range of general and selective media, including tryptic soy agar (M1938, HiMedia), Luria agar (M557, HiMedia), nutrient agar (M001, HiMedia), Reasoner’s 2A agar (M1743, HiMedia), and Burk’s medium (M707, HiMedia). Temperature tolerance was determined by incubating cultures in nutrient broth aerobically at 5°C, 10°C, 15°C, 20°C, 25°C, and 30°C for 9 days. The pH tolerance range was assessed in nutrient broth adjusted to pH values from 6 to 11 using appropriate buffering systems: acetate (pH 4–5), phosphate (pH 6–8), and glycine-NaOH (pH 9–12). Salinity tolerance was tested in nutrient broth supplemented with NaCl (0%–4%, in 0.5% increments), incubated at 30°C for 9 days. Growth under all tested conditions was monitored by measuring optical density at 600 nm (OD_600_) using a Bioscreen C microbiology growth analyzer (Oy Growth Curves AB Inc., Finland), following the manufacturer’s instructions.

Carbon source utilization and enzymatic activity were evaluated using the API 20NE, API ZYM, and API 50CH/E systems (bioMérieux, France), as well as the BIOLOG GEN III MicroPlate assay (BIOLOG Inc., USA), following the manufacturers’ protocols, with all assays incubated at 30°C.

Flexirubin-type pigment production was confirmed by applying 20% (wt/vol) potassium hydroxide (MB262, Himedia) directly to a colony and observing a color change (45). Additionally, cells grown on nutrient agar were suspended in absolute ethanol (MB228, Himedia), vortexed, and centrifuged. The UV-visible spectrum of the supernatant was recorded at 450  nm, followed by the addition of KOH to a final concentration of 1%. Any shifts in the absorption spectrum were recorded, as described in reference 42.

Antibiotic susceptibility testing (AST) was performed using commercial disc sets G-XXII-minus (OD061, Himedia) and Dodeca Universal-II (DE007, Himedia) on Mueller–Hinton agar, following standard procedures. Reference strains *Staphylococcus aureus* ATCC 25923, *Escherichia coli* ATCC 25922, and *Pseudomonas aeruginosa* ATCC 27853 were included as controls. Zones of inhibition were measured and interpreted according to the manufacturer’s criteria.

### Chemotaxonomic characterization

Whole-cell fatty acid profiles of strain UC10^T^ and the type strains of *D. linearis* (LMG 32342^T^), *D. crusticola* (DSM 16708^T^), and *D. luticola* (KCTC 52981^T^) were analyzed for comparative chemotaxonomic characterization. All strains were cultured on nutrient agar (M001, HiMedia) at pH 7.0  ±  0.2 and incubated at 30°C for 6 days. Biomass was harvested and processed following the standard protocol of Sasser (2001) using the Sherlock Microbial Identification System (version 6.1, MIDI). Briefly, total fatty acids were extracted, converted to methyl esters through saponification and methylation, and analyzed by gas chromatography. Fatty acid profiles were used to distinguish strain UC10^T^ from related *Dyadobacter* species.

Polar lipids were extracted from logarithmic-phase cultures using a methanol:chloroform:0.3% sodium chloride (2.0:1.0:0.8, vol/vol/wt) solvent system following the modified Bligh and Dyer protocol (46, 47). Lipid extracts were separated by two-dimensional thin-layer chromatography on silica gel 60 F254 plates (Merck) following the protocol outlined in reference 48. The first dimension employed chloroform:methanol:water (65:25:4, vol/vol/vol), and the second dimension used chloroform:acetic acid:methanol:water (40.5:7.5:6.0:2.0, vol/vol/vol/vol). After air-drying, plates were stained with 5% ethanolic phosphomolybdic acid (319279, Sigma-Aldrich) to visualize total lipids. Specific lipid classes were detected using ninhydrin (amino lipids [ALs]), molybdenum blue (phospholipids), Dragendorff reagent (quaternary nitrogen compounds), and α-naphthol (glycolipids), as described in reference 47.

The ability of strain UC10^T^ to utilize and degrade sodium benzoate (GRM754, HiMedia) was evaluated under both nutrient-rich and minimal conditions (49). For utilization assays, nutrient broth was supplemented with sodium benzoate at final concentrations of 100, 500, 1,000, 2,000, 5,000, and 10,000  mg/L. For degradation studies, a liquid mineral medium (LMM) was prepared containing potassium nitrate (0.5  g/L), sodium chloride (2.0  g/L), dipotassium phosphate (2.0  g/L), monopotassium phosphate (2.0  g/L), magnesium sulfate heptahydrate (0.5  g/L), calcium carbonate (0.02  g/L), and ferrous sulfate heptahydrate (0.01  g/L) (48). Sodium benzoate was added to LMM at final concentrations of 100, 500, 1,000, 2,000, or 5,000  mg/L.

Seed cultures were grown in nutrient broth containing 50  mg/L sodium benzoate and incubated at 30°C until reaching an OD_600_ of approximately 0.6. An inoculum volume of ~1% was used to initiate both nutrient broth and LMM cultures supplemented with sodium benzoate. Growth was monitored by measuring optical density at 600 nm, while sodium benzoate degradation was tracked spectrophotometrically at 230 nm over 5 days. Control conditions included uninoculated media with 50 mg/L sodium benzoate to account for abiotic degradation.

## RESULTS AND DISCUSSION

### Morphological, physiological, and biochemical characteristics

Strain UC10^T^ formed circular, convex colonies (1–2 mm in diameter) with entire margins, an opaque surface, and a sticky texture. The colonies exhibited a distinctive golden pigmentation (Fig. S1). Upon exposure to 20% potassium hydroxide, a visible shift in colony color from yellowish orange to red was observed, indicating the presence of flexirubin-type pigments. This was further confirmed spectrophotometrically by an increase in absorbance at 450 nm following KOH addition to an ethanolic extract of cell biomass. Scanning electron microscopy revealed non-flagellated, rod-shaped cells measuring 0.8–1.0  µm in width and 1.0–1.3  µm in length (Fig. S1). As with other members of the genus *Dyadobacter*, UC10^T^ was Gram stain negative and non-motile, consistent with the genus description. All strains used in the comparative analysis (*D. linearis* LMG 32342^T^, *D. crusticola* DSM 16708^T^, and *D. luticola* KCTC 52981^T^) were also Gram stain negative.

UC10^T^ grew optimally on nutrient agar in 6 days at 30°C and pH 7.0. Growth was poor on Reasoner’s 2A agar, Luria agar, and Burk’s medium and absent on tryptic soy agar. UC10ᵀ produced golden-pigmented colonies, in contrast to the bright yellow colonies of *D. linearis*, the light-yellow colonies of *D. crusticola*, and the yellow colonies of *D. luticola*. The strain grew across a wide salinity range (0%–4% NaCl), with optimal growth at 1%, whereas others showed narrower salinity ranges and lower optima (Table 1). Here, “0% NaCl” denotes growth on nutrient agar without added NaCl, though trace amounts may be present from its constituents. The pH range for UC10^T^ was 6–11, matching other reference strains, but optimal growth occurred at pH 7.0, slightly higher than *D. luticola* (pH 6.5).

**TABLE 1:** Key differential phenotypic characteristics distinguishing *D. aurulentus* UC10^T^ from closely related *Dyadobacter* species^*a*^

Characteristic	*D. aurulentus*	*D. linearis*	*D. crusticola*	*D. luticola*
Type strain	UC10^T^	AB67^T^	CP183-8^T^	T17^T^
Accession number	MCC 4019	LMG 32342	DSM 16708	KCTC 52981
Color	Golden	Bright yellow	Light yellow	Yellow
Optimum temperature (°C)	30	26	25	25
Growth pH range	6 to 11	5.5 to 10	6 to 11	6 to 11
Optimum pH	7	7	7	6.5
Salinity tolerance range (%)	0 to 4	0 to 1.5	0 to 1	0 to 2
Optimum salinity (%)	1	0.5	0.5	0.5
API 20NE				
Reduction of nitrate (NO_3_)	−	−	++	−
Fermentation of D glucose (GLU)	−	−	++	−
Hydrolysis of esculin (ESC)	++	−	++	−
Beta galactosidase (PNG)	−	−	++	++
Assimilation of D glucose (GLU)	−	−	++	−
Assimilation of L arabinose (ARA)	−	−	++	−
Assimilation of D mannose (MNE)	−	−	++	+
Assimilation of D mannitol (MAN)	−	−	−	+
Assimilation of N acetyl glucosamine (NAG)	−	−	++	−
Assimilation of maltose (MAL)	−	−	++	−
Assimilation of gluconate (GNT)	−	++	−	−
API ZYM				
Lipase (C14)	++	−	−	−
Trypsin	+	++	−	+
beta-Galactosidase	++	+	−	+
beta-Glucuronidase	−	−	−	++
alpha-Glucosidase	−	++	−	++
beta-Glucosidase	−	++	−	++
N-Acetyl-beta-glucosaminidase	−	++	−	++
alpha-Mannosidase	−	++	−	+
API 50CH/E				
D-Fructose (FRU); levulose D-arabino-hexulose	++	−	−	−
Esculin (ESC)	++	++	−	++
Maltose (MAL)	−	++	−	−
AMD starch; glycogen	−	−	++	−
BIOLOG GEN III				
Dextrin	++	++	−	−
D-Maltose	++	++	−	++
D-Trehalose	++	+	++	++
D-Cellobiose	++	−	−	++
Gentiobiose	++	++	−	++
Sucrose	++	++	−	++
D-Turanose	++	++	−	++
Stachyose	++	++	−	++
D-Raffinose	++	++	−	++
α-D-Lactose	++	++	−	++
D-Melibiose	++	++	−	++
β-Methyl-D-glucoside	++	++	−	++
D-Salicin	++	++	−	++
N-Acetyl-D-glucosamine	++	++	−	++
N-Acetyl-β-D-mannosamine	++	++	++	−
N-Acetyl-D-galactosamine	++	++	++	−
D-Mannose	++	++	−	++
D-Fructose	++	++	−	++
D-Galactose	++	−	++	++
D-Fucose	−	++	++	−
L-Fucose	++	−	++	++
Inosine	−	++	−	−
D-Sorbitol	−	++	−	−
D-Arabitol	++	++	−	−
myo-Inositol	++	++	−	−
Glycerol	−	++	−	−
D-Fructose-6-PO4	−	−	++	−
Glycyl-L-proline	−	++	−	−
L-Arginine	−	++	−	−
L-Aspartic acid	−	++	−	−
L-Glutamic acid	++	++	−	−
L-Serine	−	++	++	−
Pectin	++	++	++	−
D-Galacturonic acid	++	−	++	++
L-Galactonic acid lactone	++	−	++	++
D-Gluconic acid	++	−	++	−
D-Glucuronic acid	++	−	++	++
Quinic acid	−	++	−	−
D-Saccharic acid	−	++	−	−
L-Lactic acid	++	++	−	−
α-Keto-glutaric acid	−	−	++	−
γ-Amino-butyric acid	−	++	−	−
α-Keto-butyric acid	−	−	++	−
Acetoacetic acid	++	++	−	−
Propionic acid	−	++	−	−
Acetic acid	++	++	−	−
Formic acid	−	++	−	−
Chemical sensitivity assay	++	++	−	−
pH 6	++	++	−	++
pH 5	++	+	++	++
1% NaCl	++	−	−	++
4% NaCl	++	++	−	++
1% Sodium lactate	++	++	−	++
D-Serine	++	++	−	++
Guanidine HCl	++	++	−	++
Niaproof 4	++	++	−	++
Lithium chloride	++	++	−	++
Potassium tellurite	++	++	−	++
Sodium butyrate	++	++	−	++
Sodium bromate	++	++	−	++
Antibiotic sensitivity assay	++	++	−	++
Fusidic acid	++	++	++	−
Macrolide	++	++	++	−
Rifamycin SV	++	++	−	++
Minocycline	++	++	−	++
Lincomycin	++	−	++	++
Vancomycin	−	++	++	−
Nalidixic acid	++	−	++	++
Aztreonam	−	++	−	−

^*a*^ All data were obtained in this study under identical conditions for comparative purposes. ++, positive; +, weakly positive; −, negative; BL, borderline; ESC, esculin; MAL, maltose.

Growth temperature profiling revealed that UC10^T^ tolerated a broad temperature range (5°C–30°C), showing sustained growth at 15°C and limited but consistent growth at 5°C–10°C after 7 days (Fig. S2). Optimal growth occurred at 30°C, whereas *D. crusticola* and *D. luticola* preferred 25°C. Unlike *D. linearis*, which grew up to 37°C, UC10^T^ failed to grow at this temperature, consistent with its cold-adaptive phenotype. These observations suggest that while UC10^T^ thrives under mesophilic laboratory conditions, it retains functional capacity for cold tolerance, potentially reflective of its adaptation to fluctuating temperature regimes in high-altitude agricultural soils. Further *in situ* studies would help define its ecological performance relative to ambient annual temperature cycles at its source location (Gangotri; 3,415  m a.s.l.).

API 20NE, API ZYM, and API 50CH/E tests revealed unique metabolic and enzymatic signatures that distinguish UC10^T^ from its closest relatives (Table 1). UC10^T^ lacked nitrate reduction and glucose fermentation activities, both present in *D. crusticola*. Esculin hydrolysis was observed in UC10^T^ and *D. crusticola*, but not in *D. linearis* or *D. luticola*. In API ZYM, UC10^T^ exhibited strong lipase (C14) and β-galactosidase activity, traits absent in *D. linearis* and *D. crusticola* but partially shared with *D. luticola*. Conversely, UC10^T^ lacked α- and β-glucosidase activities, which were prominent in the other strains, providing further biochemical discrimination. In API 50CH/E assays, UC10^T^ showed limited carbohydrate assimilation, notably failing to utilize D-glucose, L-arabinose, D-mannose, D-mannitol, N-acetyl-glucosamine, maltose, or gluconate, in contrast to broader substrate usage in *D. crusticola*. This minimalistic profile aligns with an ecophysiological strategy favoring low-nutrient or specialized environments.

BIOLOG GEN III profiling revealed extensive differences in carbon utilization, chemical tolerance, and antibiotic resistance among the four *Dyadobacter* strains (Table 1; Table S1). UC10^T^ and *D. linearis* shared the ability to metabolize dextrin, D-maltose, sucrose, D-raffinose, α-D-lactose, D-melibiose, and D-salicin. However, UC10^T^ did not utilize N-acetyl-β-D-mannosamine, N-acetyl-D-galactosamine, or pectin, substrates assimilated by *D. luticola*. D-galactose was utilized by UC10^T^ and *D. luticola* but not by *D. linearis*. In contrast, D-fucose was metabolized by *D. linearis* and *D. crusticola* but not by UC10^T^. *D. linearis* also uniquely assimilated inosine and D-sorbitol, indicating niche-specific metabolic divergence.

Chemical sensitivity patterns revealed notable interspecies variation. UC10^T^ tolerated up to 1% NaCl and pH 6 but was sensitive to acidic conditions (pH 5). *D. linearis* showed superior resistance to environmental stressors, including guanidine hydrochloride, Niaproof 4, lithium chloride, and sodium bromate. *D. luticola* displayed the highest halotolerance (4% NaCl) and was the only strain tolerant to potassium tellurite. Antibiotic resistance profiling (BIOLOG GEN III) further highlighted functional distinctions. UC10^T^ and *D. linearis* shared resistance to rifamycin SV, lincomycin, vancomycin, nalidixic acid, and aztreonam. *D. linearis* and *D. luticola* also exhibited resistance to macrolides and fusidic acid, while *D. crusticola* remained broadly susceptible.

*In vitro* AST using commercial disks confirmed a broad susceptibility profile for UC10^T^ (Table S2). Strong inhibition was observed with tetracycline (40 mm), co-trimoxazole (34/27 mm), amikacin (32/33 mm), norfloxacin (40 mm), ciprofloxacin (38 mm), and furazolidone (33 mm). Moderate sensitivity was noted for chloramphenicol, kanamycin, amoxicillin, ceftriaxone, and colistin. Resistance was observed against streptomycin (10 mm) and gentamicin (13–17 mm), and intermediate responses to ampicillin, amoxicillin-clavulanic acid, netillin, and cefotaxime suggest possible β-lactam resistance mechanisms (Table S2). Reference strain responses aligned with CLSI guidelines.

Overall, the results reveal UC10^T^ to be phenotypically distinct from closely related *Dyadobacter* spp., particularly in pigmentation, salinity and temperature tolerance, enzymatic activities, and carbon source utilization. Its moderate halotolerance, cold adaptability, and streamlined substrate preferences may reflect evolutionary pressures in nutrient-poor, seasonally cold, high-altitude agricultural soils. These adaptations, along with distinct chemotaxonomic traits and genomic evidence, support the classification of UC10^T^ as a novel species within the genus *Dyadobacter*.

### Chemotaxonomic characterization

Strain UC10^T^ exhibited a distinct fatty acid composition compared to its closest relatives (*D. linearis* LMG 32342^T^, *D. crusticola* DSM 16708^T^, and *D. luticola* KCTC 52981^T^), as summarized in Table S3. UC10^T^, isolated from cold, high-altitude Himalayan soil, was characterized by a high proportion of C_16:0_ (28.9%) and a substantial unsaturated component represented by summed feature 3 (41.2%, comprising C_16:1_  ω7c/_C16:1_ ω6c). Additional fatty acids included C_14:0_ iso (7.7%), C_13:0_ (4.1%), C_15:1_ iso G (4.9%), and C_16:1_  ω5c (5.4%). In contrast, *D. crusticola*, isolated from arid soil crusts, contained lower C_16:0_ (21.7%) and elevated C_14:0_ iso (14.3%), along with summed feature 9 (1.6%, C_16:0_ 10-methyl/_C17:1_ iso ω9c), likely contributing to membrane stabilization under desiccation and thermal stress. *D. luticola*, from sewage sediment, displayed a branched fatty acid-rich profile dominated by C_15:0_ iso (26.2%) and C_16:0_ (18.7%), as well as a moderate unsaturated fraction (summed feature 3, 33.6%), suggesting adaptation to chemically dynamic, nutrient-rich environments. Among the four strains, *D. linearis* exhibited the most divergent FAME profile. It contained a markedly lower proportion of C_16:0_ (4.7%) but higher levels of C_15:0_ iso (18.8%), C_17:0_ 3OH (12.2%), and C_15:0_ 3OH (2.8%), none of which were detected in UC10^T^. Summed feature 9 (2.7%) was also unique to *D. linearis* and absent in UC10^T^. Collectively, these fatty acid profiles reflect environmental specialization: high C_16:0_ and unsaturated fatty acid content in UC10^T^ is consistent with membrane fluidity adaptations to freezing temperatures, whereas the profiles of the other strains reflect tolerance to desiccation (*D. crusticola*), high organic loads (*D. luticola*), or aquatic conditions (*D. linearis*), aligning with their respective isolation sources.

Polar lipid analysis further supported the taxonomic distinction of UC10^T^. The major polar lipid detected was phosphatidylethanolamine (PE), accompanied by ALs, amino-phospholipids, and several unidentified polar lipids (ULs). Notably, phosphatidylglycerol, present in *D. linearis*, was absent in UC10^T^. Additionally, *D. linearis* possessed unique glycolipids (GL1) and several distinct unidentified lipids (UL1–UL5), which were not detected in UC10^T^. *D. luticola* and *D. crusticola* also exhibited PE as the dominant lipid, similar to UC10^T^. However, *D. luticola* was distinguished by a prominent unknown polar lipid (L2), only faintly detected in other strains. While diphosphatidylglycerol was absent in all tested strains, minor variations in unidentified lipid fractions were observed across species. These lipid profiles suggest niche-specific membrane adaptations and contribute additional chemotaxonomic resolution. A detailed lipid component comparison is provided in Fig. S3. The combined FAME and polar lipid data confirm that UC10^T^ possesses a distinct membrane composition that likely contributes to its ability to thrive in high-altitude, cold, and oligotrophic soil conditions. These membrane-level adaptations, together with other physiological traits, support its recognition as a novel species within the genus *Dyadobacter*.

### Phylogenetic analysis and overall genome relatedness index

**Fig 1:**
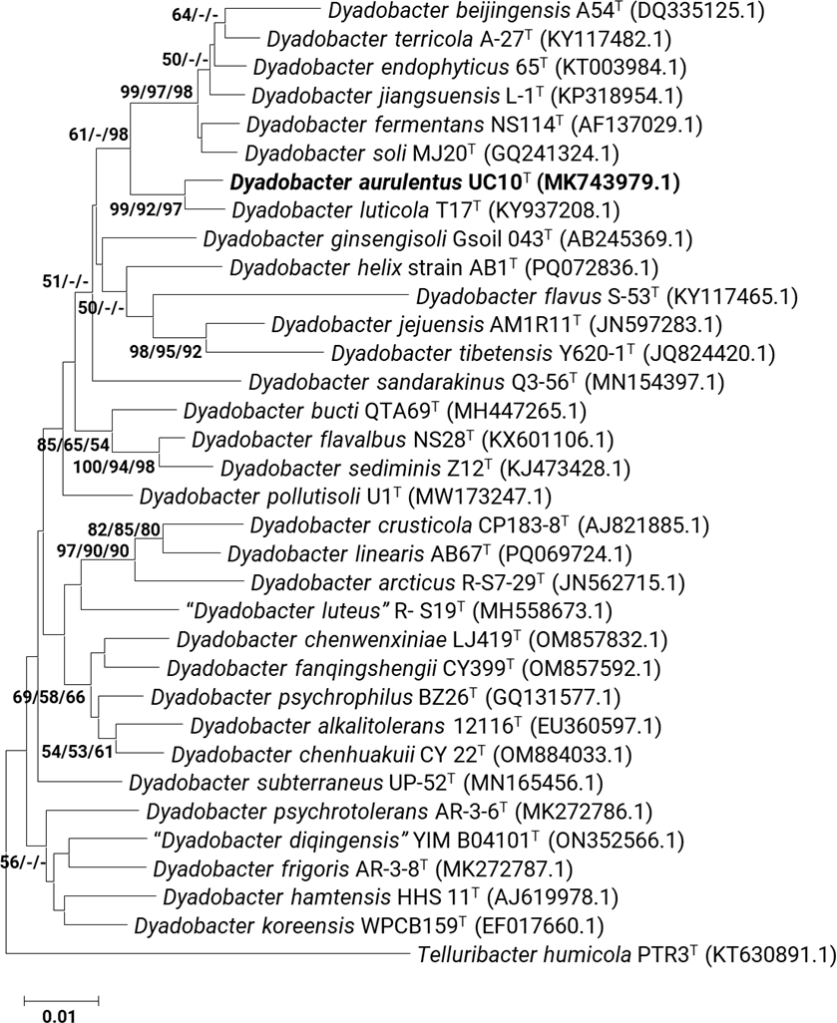
A phylogenetic tree, developed from 16S rRNA gene sequences, illustrates the relationship between *Dyadobacter aurulentus* UC10^T^ (MK743979) and closely related members. The tree was constructed employing neighbor-joining (NJ), maximum-likelihood (ML), and maximum parsimony (MP) methods using MEGA 11 Bootstrap analysis, executed with 1,000 replicates, consistently supported all three methodologies. The core framework presented above relied on the ML-generated tree under the Kimura 2-parameter model, with subsequent integration of bootstrap values for all methods. Nodal figures on branches represent percentage support from NJ, ML, and MP analyses, respectively. This data set encompassed 1,342 positions, utilizing 16S rRNA gene sequence of *Telluribacter humicola* strain PTR3^T^ (KT630891) as the outgroup. A bar indicates nucleotide substitutions per site, with tree display excluding bootstrap values below 50.

The phylogenetic position of strain UC10ᵀ was inferred from its nearly full-length 16S rRNA gene sequence (MK743979), which showed 98.95% similarity with *D. luticola* KCTC 52981ᵀ (KY937208), followed by 97.68% with *D. crusticola* DSM 16708ᵀ and 97.40% with *D. koreensis* DSM 19938ᵀ. In 16S rRNA gene-based phylogenetic analyses, UC10^T^ clustered in a monophyletic clade with *D. luticola* KCTC 52981^T^, suggesting a close but separate lineage within the genus *Dyadobacter* (Fig. 1).

Illumina sequencing of UC10^T^ yielded 1,608,277 paired-end reads (150 × 2 bp), and Oxford Nanopore sequencing produced 64,405 long reads ranging from 130 bp to 30.39 kb. A hybrid assembly generated a draft genome comprising 13 contigs, with a total size of 6.92 Mb, an N50 of 6.9 Mb, and the largest contig measuring 6,918,535 bp. Genome coverage averaged 102×, with an estimated completeness of 96.75% and contamination of 3.79% (CheckM). The genome has been deposited in GenBank under accession number VSRN01. The Sanger-generated 16S rRNA gene sequence (MK743979; 1,460 bp) was found to be identical to both copies extracted from the genome sequence (locus tags FXO21_07285 and FXO21_11025, each 1517 bp), confirming the authenticity of the genome assembly. When compared to other members of the genus, UC10^T^ possessed the largest genome, followed by *D. linearis* and *D. luticola* (6.4 Mb each), and *D. crusticola* (6.2 Mb). Reported genome sizes within the genus *Dyadobacter* range from approximately 5.3 to 9.2  Mb based on publicly available assemblies in the NCBI Genome Browser. G + C contents among the four strains were broadly similar, with UC10^T^ T at 46.5 mol%, comparable to *D. linearis* LMG 32342^T^ (46.07%), *D. crusticola* DSM 16708^T^ (46.73%), and *D. luticola* KCTC 52981^T^ (47%).

The genome of strain UC10^T^ encodes the highest number of predicted protein-coding sequences (5,695) among the four compared *Dyadobacter* species, followed by *D. linearis* LMG 32342^T^ (5,427), *D. luticola* KCTC 52981^T^ (5,422), and *D. crusticola* DSM 16708^T^ (5,251), based on annotations from the NCBI RefSeq database as of 16 December 2024. While *D. linearis* possesses a slightly smaller genome (6.4 Mb) than UC10^T^ (6.92 Mb), it harbors fewer coding genes, suggesting potential functional genome expansion in UC10^T^. The number of RNA genes also varied across genomes. UC10^T^ encoded 9 rRNA genes and 41 tRNA genes, higher than *D. linearis* (8 rRNA genes and 38 tRNA genes), *D. crusticola* (6 rRNA genes and 36 tRNA genes), and *D. luticola* (7 rRNA genes and 39 tRNA genes) (Table 2). These differences in genome architecture, particularly in gene number and RNA content, may reflect adaptive genomic plasticity in UC10^T^, possibly linked to environmental stress responses, metabolic versatility, or ecological specialization.

**TABLE 2:** Assembly and annotation statistics of *Dyadobacter aurulentus* UC10^T^ genome (VSRN01) and its closest phylogenetic neighbor *D. crusticola* CP183-8^T^ (JNJB01) and *D. luticola* T17^T^ (VCEJ01)^*b*^

Characteristic	*D. aurulentus*	*D. linearis*	*D. crusticola*	*D. luticola*
Strain	UC10^T^	AB67^T^	CP183-8^T^	T17^T^
Culture collection	MCC 4019	LMG 32342	DSM 16708	KCTC 52981
Accession no. (16S rRNA)	MK743979	PQ069724	AJ821885	KY937208
% Identity (16S rRNA)	–^*c*^	96.95	95.87	98.95
Genome accession no.	VSRN01	CAJRAU01	JNJB01	VCEJ01
No. of contigs	13	27	45	16
Genome size (Mb)	6.9	6.4	6.2	6.4
G+C (%)	46.5	46.07	46.73	47
dDDH (%)	–	23.7	19.17	18.5
ANI (%)	–	80.63	76.55	74.33
EzAAI (%)^*a*^	–	87.50	82.6	78.71
CDS	5,677	5,427	5,217	5,406
rRNA	9	8	6	7
tRNA	41	38	36	39

^*a*^ EzAAI (%): average amino acid identity (AAI) values calculated using the EzAAI tool available on the EzBioCloud platform, which aligns predicted coding sequences (CDSs) to compute average amino acid identity (29). For comparison, AAI values calculated using the Kostas Lab AAI calculator were 81.2% (*D. crusticola*) and 76.8% (*D. luticola*), which uses reciprocal best hits to assess the average identity of homologous proteins between genomes (30).

^*b*^ Additionally, see Table S4 for pairwise ANI and AAI values and Table S5 for digital DNA–DNA hybridization (dDDH) results between UC10^T^ and all published *Dyadobacter* species.

^*c*^ –, not applicable.

**Fig 2:**
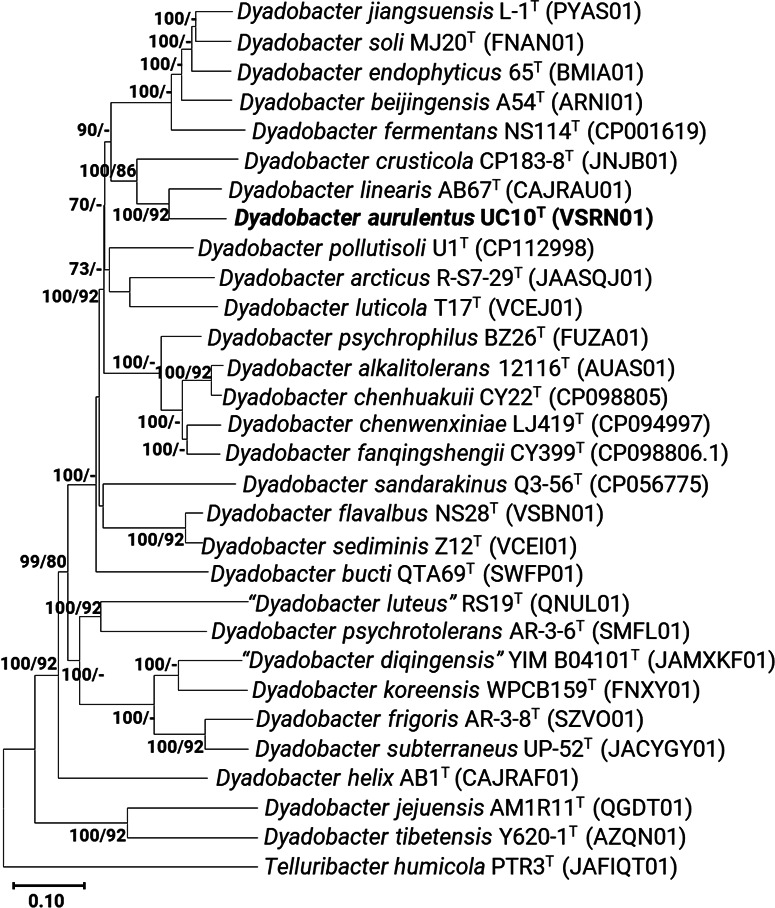
A combined genome phylogenetic tree of *Dyadobacter aurulentus* UC10^T^ (VSRN01) and related *Dyadobacter* spp. The tree was constructed using orthologous gene and protein sequences obtained through BPGA and UBCG pipelines. The BPGA tree was generated using the neighbor-joining method with 1,000 bootstrap replicates, while UBCG produced a separate tree based on concatenated and aligned nucleotide sequences. The backbone topology shown reflects the BPGA-derived tree, with bootstrap values at each node indicating support from both BPGA and UBCG analyses, respectively. *Telluribacter humicola* PTR3^T^ (JAFIQT01) was used as the outgroup. The scale bar represents the number of substitutions per site.

Whole-genome phylogenetic reconstruction based on orthologous protein and core gene alignments revealed a different pattern. UC10^T^ formed a distinct monophyletic branch with *D. linearis* LMG 32342^T^ (CAJRAU01), indicating it as the closest genome-level relative (Fig. 2). This relationship was reinforced by overall genomic relatedness indices. UC10^T^ shared 80.63% ANI and 23.7% dDDH with *D. linearis*, both values well below the accepted species thresholds of 95%–96% ANI and 70% dDDH. Comparatively lower ANI and dDDH values were observed with *D. crusticola* DSM 16708^T^ (76.55% ANI and 19.17% dDDH) *D. luticola* KCTC 52981^T^ (74.33% ANI and 18.5% dDDH) (Table 2; Tables S4 and S5), further substantiating the genomic distinctiveness of UC10^T^.

AAI analysis yielded similar results. UC10^T^ showed the highest AAI value (87.50%) with *D. linearis*, followed by 82.6% with *D. crusticola* and 78.71% with *D. luticola* (Table 2). All values fall below the typical interspecies AAI threshold of <95%, providing additional support for species-level separation (Table S4).

Together, the 16S rRNA gene-based phylogeny, genome-based tree topology, ANI, dDDH, AAI metrics, and genome composition data all converge to indicate that strain UC10^T^ represents a genomically and phylogenetically distinct lineage within the genus *Dyadobacter*. These findings justify its recognition as a novel species and validate the inclusion of *D. linearis* LMG 32342^T^, *D. crusticola* DSM 16708^T^, and *D. luticola* KCTC 52981^T^ as reference comparators in this polyphasic taxonomic study.

### Genome features

COG annotation revealed that strain UC10^T^ possesses the highest number of functionally categorized genes across several major biological processes among the compared *Dyadobacter* spp. Notably, UC10^T^ harbors 396 genes associated with transcription, 376 genes related to carbohydrate transport and metabolism, and 396 genes involved in cell wall/membrane/envelope biogenesis. These values exceed those found in its closest genomic relative, *D. linearis* LMG 32342^T^, which contains 361, 327, and 372 genes in the respective categories. These differences suggest subtle but potentially significant divergences in metabolic breadth, regulatory complexity, and membrane architecture between the strains.

The unique genomic potential of UC10^T^ is further underscored by a comparative Venn diagram analysis (Fig. S4), which shows UC10^T^ encodes 111 strain-specific genes not found in *D. linearis*, *D. crusticola*, or *D. luticola*. This number is notably higher than that observed for *D. crusticola* (100), *D. luticola* (69), and *D. linearis* (37), pointing to the presence of novel genetic elements potentially associated with adaptation to its high-altitude, cold-soil niche. Intriguingly, the genome of UC10^T^ also contains 1,137 genes assigned to COG category “S” (function unknown), the highest among the compared genomes. This abundance of hypothetical or uncharacterized proteins suggests the existence of unexplored metabolic pathways or regulatory networks that may play roles in environmental sensing, stress response, or niche-specific interactions. The identification and functional characterization of these genes may offer new insights into microbial adaptation under suboptimal temperature and nutrient-limited conditions.

Despite this diversity, all four strains share a core genome of 1,539 orthologous genes, reflecting evolutionary cohesion within the *Dyadobacter* genus. However, the extended accessory and strain-specific gene content in UC10^T^ contributes to its distinct functional profile and reinforces its separation from established species. These genomic patterns, in combination with phenotypic and ecological data, support its recognition as a novel species.

Genome analysis of UC10^T^ revealed two distinct cold shock protein (CSP) genes: WP_026629450.1 and WP_031526203.1, both assigned to KEGG Orthology entry K03704. In contrast, *D. linearis* LMG 32342^T^ encoded only a single CSP (WP_031526203.1). Although these CSPs are included in the KEGG BRITE functional classification, they are not linked to any complete cold adaptation pathway module, suggesting that while they are functionally significant, they are not part of a defined modular system, or that their roles within such modules remain uncharacterized (50, 51). This partial modular mapping reflects the complexity of bacterial cold response, which often involves CSP-independent mechanisms. In UC10^T^, multiple additional genomic features point to a multifaceted cold adaptation strategy. These include fatty acid desaturases (WP_149638574.1 and WP_149639218.1), which help maintain membrane fluidity at low temperatures, cold-inducible chaperones (WP_149638781.1 and WP_149639319.1) that stabilize protein conformation, and RNA helicases (WP_149639405.1 and WP_149638422.1), which support translation efficiency by resolving secondary mRNA structures under cold stress. The presence of these genes suggests that UC10^T^ is equipped with a robust and redundant cold response system that acts at the level of transcription, translation, and membrane homeostasis.

Consistent with these genomic predictions, UC10^T^ displayed broad temperature tolerance, growing from 5°C to 30°C, with optimal growth at 30°C. Its ability to grow, albeit slowly, at 5°C–10°C without classical psychrophilic traits (preference for ≤15°C) indicates a cold-adaptive phenotype adapted to variable thermal regimes. The genomic data suggest that this flexibility is not solely dependent on CSPs but is reinforced by auxiliary regulatory and structural adaptations, including putative two-component systems (e.g., DesK–DesR) and osmo-protectant pathways (e.g., trehalose synthesis) described in other cold-adaptive or psychrotolerant bacteria (51). This integrated response system likely contributes to ecological persistence in cold, high-altitude agricultural soils, where temperatures fluctuate sharply across seasons and diurnal cycles. Interestingly, this growth pattern aligns with the Ratkowsky biokinetic model, which predicts that bacterial growth optima are typically higher than the ambient environmental temperature, an evolutionary strategy that allows rapid exploitation of transient favorable conditions (52). Such thermal flexibility may reflect a general adaptive principle among cold-soil bacteria, including UC10^T^. Elucidating these regulatory pathways may provide insights into cold stress resilience mechanisms and open avenues for identifying cold-active biomolecules for environmental or industrial applications.

Phenotypically, UC10^T^ and the three reference *Dyadobacter* strains were non-motile, consistent with known traits of the genus. However, genomic analysis revealed the presence of multiple genes encoding components of the flagellum-independent gliding motility system, including *gldN* (WP_031529436.1), *gldE* (WP_149639807.1), *gldL* (WP_149639947.1), *gldM* (WP_149639918.1), and *gldC* (WP_149639814.1), and associated lipoproteins *gldD* (WP_149639714.1), *gldJ* (WP_149639807.1), *gldK* (WP_149639818.1), and *gldH* (WP_149639910.1). ABC transporter components *gldA* (WP_149639855.1) and *gldF* (WP_149639631.1) were also identified, indicating the presence of a largely intact type IX secretion system (T9SS), commonly involved in surface motility and protein export in Bacteroidota (53). Additionally, key surface adhesin genes, *sprB* (WP_149639919.1) and *remA* (WP_149639927.1), were present too, in UC10^T^. These adhesins are essential for contact-dependent movement (54, 55). Despite the genomic completeness of this machinery, UC10^T^ displays no gliding motility under standard laboratory conditions. This incongruity may result from regulatory suppression, impaired expression or localization of adhesins, lack of post-translational activation, or environmental cues required for motility induction, such as nutrient limitation, soft agar substrates, or other unknown reasons. Further experimental investigation, such as transcript profiling, surface protein localization, or condition-specific motility assays, would be required to resolve this discrepancy. However, such analyses are beyond the scope of the present study.

KEGG annotations suggest that strain UC10^T^ encodes multiple genes involved in carbohydrate metabolism, despite its limited *in vitro* assimilation of substrates such as glucose, sorbitol, mannitol, and glycerol (Table 1; Table S1). Among these are genes such as fructose-1,6-bisphosphatase I (FBPase, K03841) and maltose O-acetyltransferase (MAT, K00661), which are associated with gluconeogenesis and sugar modification, respectively. The genome also harbors multiple DeoR/GlpR family transcriptional regulators (e.g., WP_149638170.1 and WP_149639278.1), known to control sugar transport and metabolic gene expression under fluctuating nutrient conditions (56). Furthermore, no evidence was found in the protein annotations for putative glucose uptake proteins, glucose–fructose oxidoreductase, or maltose phosphorylase, suggesting these genes may be absent, poorly annotated, or conditionally expressed. The mismatch between genomic predictions and experimentally observed sugar assimilation likely reflects tight regulatory control, environmentally triggered gene expression, or incomplete transporter systems. These features may represent an ecological strategy favoring metabolic economy and selective substrate use in high-altitude, low-nutrient environments. UC10^T^ appears to be adapted to the oligotrophic and cold conditions of Himalayan farmland soils, where a streamlined and regulated metabolic response would offer a selective advantage.

In high-altitude soils, exposure to plant-derived aromatics and anthropogenic inputs can exert selective pressure on microbial communities to evolve pathways for aromatic compound transformation. The genome of strain UC10^T^ encodes a partial yet functionally coherent set of genes associated with benzoate degradation, including acyl-CoA dehydrogenases (WP_149641180.1 and WP_149641843.1), acetyl-CoA C-acyltransferases (WP_149641337.1 and WP_149640196.1), alpha/beta hydrolases (WP_149641599.1), and 4-oxalocrotonate tautomerase (WP_149638702.1), as well as VOC and RraA family proteins (WP_149642814.1 and WP_149643117.1). These enzymes suggest a modular, non-canonical pathway for processing benzoate and related aromatic intermediates (57, 58). This genomic potential was corroborated by *in vitro* assays, where UC10^T^ exhibited growth across a range of sodium benzoate concentrations, with the highest OD_600_ recorded at 100 mg/L, and actively degraded the compound in mineral medium, particularly at initial concentrations of 2,000 and 5,000 mg/L (Fig. S5). These results confirm that UC10^T^ can utilize sodium benzoate as a sole carbon and energy source under laboratory conditions.

Together, the genomic and phenotypic evidence indicates that UC10^T^ harbors a non-canonical but operational benzoate degradation pathway, likely adapted for transformation of aromatic substrates under nutrient-limited conditions. This catabolic capacity may contribute to ecological resilience in high-altitude agricultural soils enriched with plant-derived aromatics or xenobiotics and highlights potential for bioremediation applications targeting benzoate and related pollutants.

Environmental bacteria exposed to naturally occurring antibiotics and other stressors can shape their genomic repertoire for antimicrobial resistance and secondary metabolite production. In this context, genome mining of strain UC10^T^ revealed several genes linked to both antibiotic resistance and antimicrobial compound biosynthesis, suggesting its potential role in microbial competition and resilience in high-altitude soil environments. In strain UC10^T^, genome analysis revealed multiple genes associated with β-lactam antibiotic resistance, including several BlaI/MecI/CopY family transcriptional regulators (WP_149639011, WP_149640165, and WP_149643120), known to regulate β-lactamase expression (59, 60). The genome also encodes penicillin-binding proteins (PBPs) (WP_149640026 and WP_149640227), transglycosylase domain-containing proteins (WP_149638930 and WP_149642110), and a subclass B1 metallo-β-lactamase (WP_149641631), all contributing to β-lactam hydrolysis and peptidoglycan remodeling (61). Efflux-mediated resistance is suggested by the presence of a diverse set of resistance–nodulation–division (RND) efflux pump components, including periplasmic adaptors (e.g., WP_149640326 and WP_149641644) and TolC-family outer membrane proteins (WP_149640325, WP_149640973), known to expel a wide range of antibiotics.

These genomic predictions were validated by antimicrobial susceptibility testing (AST). UC10^T^ showed resistance to ampicillin and amoxicillin, consistent with its β-lactamase and PBP repertoire (Table S2). It also exhibited partial resistance to tetracycline, streptomycin, and co-trimoxazole, which likely reflects the action of RND-type efflux pumps and TolC channels. No resistance genes were detected for aminoglycosides (e.g., gentamicin), fluoroquinolones (e.g., ciprofloxacin), or other antibiotic classes to which UC10^T^ was phenotypically sensitive, supporting the specificity of genomic predictions (Table S2). In addition to resistance traits, UC10^T^ harbors a biosynthetic gene cluster for class I lanthipeptides, a group of ribosomally synthesized antimicrobial peptides with therapeutic potential. This cluster includes lantibiotic biosynthesis proteins (WP_149638273–WP_149638275) and a LanC-type lanthionine synthetase (WP_149638277.1), absent in the genomes of *D. linearis* and *D. crusticola*. Lanthipeptides are of growing interest as alternative antimicrobials, particularly against multidrug-resistant pathogens.

Altogether, the AST results and genomic findings in UC10^T^ demonstrate a congruent resistance profile that combines target-site modifications, enzymatic inactivation, and efflux-based mechanisms, while also revealing biosynthetic potential for antimicrobial compound production. These traits underscore both the ecological competitiveness and potential applied value of UC10^T^ in environments with complex antibiotic landscapes.

In addition to experimentally validated traits, the genome of UC10^T^ encodes several other features indicative of broad ecological adaptability. Although these were not explored functionally in this study, their presence offers insight into broader metabolic and stress response potential. For example, UC10^T^ harbors 19 genes related to quorum sensing and peptide secretion, including a lantibiotic biosynthesis protein (WP_149638272) and the two-component regulator *FusR* (WP_149641494), both associated with population-density sensing (62, 63). Genes involved in peptide export, such as a *SecD*/*SecF* fusion protein (WP_149639775), membrane fusion protein (WP_149639039), and ABC transporter (WP_192579356), suggest active roles in signaling and surface-associated interactions (64).

UC10^T^ encodes genes involved in nitrate assimilation, which may allow it to thrive in nitrogen-limited soils. These include a NarK family MFS transporter (WP_149640743.1) and nitrite reductase subunits NirD (WP_149640739.1), NirB (WP_149640740.1), and an additional copy (WP_149640744.1). These genes participate in the conversion of nitrate to ammonia, an essential step in assimilatory nitrogen metabolism (65). The presence of these components highlights potential for flexible nitrogen acquisition in variable or nutrient-depleted environments.

UC10^T^ encodes multiple heavy metal resistance genes, including *CusA*/*CzcA* transporters (WP_149638583.1, WP_149638619.1) for copper, zinc, and cobalt efflux (66), and arsenite efflux pumps (WP_149638335.1, WP_149638336.1) for arsenic detoxification (67). While *D. linearis* also harbors a mercury transporter (WP_215236589.1), this was not detected in UC10^T^ or *D. crusticola*. The metal resistance profile of UC10^T^ suggests potential for survival in contaminated soils and application in bioremediation.

Together, these additional genomic features highlight the potential of UC10^T^ for surface colonization, nutrient adaptation, and metal stress resilience, further supporting its ecological versatility and functional relevance in high-altitude, cold-soil environments.

### Conclusion

This study presents a comprehensive taxonomic and functional characterization of strain UC10^T^, isolated from high-altitude agricultural soils in the Western Himalayas. Integrating phenotypic, chemotaxonomic, and genomic data, we establish UC10^T^ as a novel species within the genus *Dyadobacter*, for which the name *Dyadobacter aurulentus* sp. nov. is proposed. All overall genome relatedness indices (including ANI (80.63% with *D. linearis*), AAI (87.50%), and dDDH (23.7%), fall well below established species delineation thresholds, providing robust genomic evidence for species-level distinction. Supporting this conclusion, chemotaxonomic and physiological analyses provided additional lines of evidence for species delineation. Fatty acid and polar lipid profiles, along with distinct substrate utilization and stress tolerance traits, differentiated UC10^T^ from its closest relatives.

The genome of UC10^T^ reflects a broad ecological and functional repertoire. Key features include genes associated with cold adaptation (cold shock proteins, fatty acid desaturases), partial benzoate degradation pathways, quorum sensing, nitrate assimilation, and heavy metal resistance. Notably, *in vitro* assays confirmed the ability to degrade sodium benzoate and grow at 5–30°C, validating genomic predictions related to aromatic compound catabolism and cold-adaptive nature.

UC10^T^ also harbors multiple antibiotic resistance determinants, including β-lactamases, BlaI/MecI regulators, and RND-type efflux systems, with *in vitro* resistance observed against ampicillin, amoxicillin, tetracycline, and streptomycin. Importantly, it encodes a biosynthetic cluster for class I lanthipeptides, ribosomally synthesized antimicrobial peptides with potential as novel therapeutics, highlighting its biotechnological relevance. Together, these features suggest the capacity of UC10^T^ to thrive in cold, oligotrophic, and potentially contaminated soils and support its use in bioremediation, low-temperature biodegradation, and cold-region bioaugmentation.

This work expands our understanding of the genus *Dyadobacter*, revealing a novel member, *Dyadobacter aurulentus* sp. nov., adapted to cold agricultural soils and capable of aromatic compound degradation at low temperatures. Representing one of the cold-adaptive, aromatic compound-degrading species described from Himalayan farmland soils, *D. aurulentus* broadens the phylogenetic and functional diversity of the genus. The integration of genomic and phenotypic data in this study highlights the value of genome-enabled polyphasic taxonomy in uncovering both the identity and ecological roles of novel microorganisms. By linking genomic insights with experimental validation, this work demonstrates how integrative microbiology can reveal functionally versatile environmental bacteria with potential applications in environmental and industrial biotechnology.

### Description of *Dyadobacter aurulentus* sp. nov.

#### *Dyadobacter aurulentus* sp. nov. (au.ru.len’tus. L. masc. adj. aurulentus, golden colored), referring to the color of colony

Colonies are golden in color with an entire margin and convex elevation, and cells are Gram stain-negative, catalase- and oxidase-positive, non-motile, short rods measuring 0.8–1.0 µm in width and 1.0–1.5 µm in length. Growth occurs optimally on nutrient agar within 5–6 days at 30°C, at pH 7, and 1% NaCl. It hydrolyzes esculin and assimilates a broad range of carbohydrates (including dextrin, D-maltose, D-cellobiose, gentiobiose, sucrose, turanose, stachyose, D-raffinose, α-D-lactose, D-melibiose, β-methyl-D-glucoside, D-salicin, N-acetyl-D-glucosamine, N-acetyl-β-D-mannosamine, N-acetyl-D-galactosamine, D-mannose, D-fructose, D-galactose, D-arabitol, and myo-inositol). It also utilizes L-fucose, L-glutamic acid, pectin, D-galacturonic acid, L-galactonic acid lactone, D-gluconic acid, D-glucuronic acid, L-lactic acid, acetoacetic acid, and acetic acid. It tolerates 1% sodium lactate; is sensitive to fusidic acid, troleandomycin, minocycline, lithium chloride, potassium tellurite, and sodium butyrate, guanidine HCl, Niaproof 4, and sodium bromate; contains major fatty acids, C_16:00_ (28.9%) and a significant presence of C_14:0_ iso (7.7%) and C_13:00_ (4.1%) with a summed feature 3 (41.2%) that comprises C_16:1_ ω7c/C_16:1_ ω6c. Polar lipids, PE, amino lipids, amino phospholipids, and two unknown lipids. The G + C content of type strain UC10^T^ is 46.5%.

The type strain UC10^T^ (=MCC 4019^T^ = KCTC 72,455^T^ = JCM 34514^T^) was isolated from high-altitude farmland soil from Gangotri (India) region of Western Himalaya.

## Data Availability

The GenBank/EMBL/DDBJ accession numbers for the reference 16S rRNA gene sequence and the whole genome of strain UC10^T^ are MK743979 and VSRN01 (assembly GCA_008369915.1), respectively. Sequence Read Archive data for Illumina and Oxford Nanopore Technologies (ONT) sequencing of UC10^T^ can be found under GenBank accession numbers SRR29320013 (Illumina) and SRR29321024 (ONT). This genome was submitted under BioProject ID PRJNA561267 and BioSample ID SAMN12612869.

## SUPPLEMENTAL MATERIAL

Supplemental material spectrum.01145-25-s0001.docx Fig. S1 to S3; Tables S1 to S3.

Table S4 spectrum.01145-25-s0002.xlsx Pairwise genomic relatedness between *Dyadobacter aurulentus* UC10ᵀ and recognized Dyadobacter species based on average nucleotide identity and average amino acid identity.

Table S5 spectrum.01145-25-s0003.xlsx Pairwise genomic relatedness between *Dyadobacter aurulentus* UC10ᵀ and recognized Dyadobacter species based on digital DNA-DNA hybridization.

ASM does not own the copyrights to Supplemental Material that may be linked to, or accessed through, an article. The authors have granted ASM a non-exclusive, world-wide license to publish the Supplemental Material files. Please contact the corresponding author directly for reuse.
