# Supplementary material for: Description of high-altitude, cold-adaptive, metabolically versatile Dyadobacter aurulentus sp. nov. isolated from Western Himalayan farmland soils
Source: Microbiol Spectr. 2025 Oct 9;13(11):e01145-25. doi: 10.1128/spectrum.01145-25 (PMC12584681; doi:10.1128/spectrum.01145-25)
Supplement: Supplemental material — Fig. S1 to S3; Tables S1 to S3. [file spectrum.01145-25-s0001.docx]

**Table S1**. List of all traits which are not included in differential phenotypic characteristics Table 1 of *Dyadobacter aurulentus* UC10^T^ (MCC 4019) and its closest relatives *D. linearis,* AB67^T^ (LMG 32342), *D. crusticola* CP183-8^T^ (DSM 16708) and *D. luticola* T17^T^ (KCTC 52981). All data were obtained from this study.

| **All strains tested positive for** | **All strains tested negative for** |
| --- | --- |
|  | Gram Staining |
| **API 20NE** |  |
| None | Production of Indol, hydrolysis of Arginine dihydrolase, Urease and Gelatine. Assimilation of capric acid, Adapic acid, malic acid, citric acid and phenyl acetic acid. |
| **API 20E** |  |
| None | All listed |
| **API ZYM** |  |
| Alkaline phosphatase, Esterase (C4), Esterase Lipase (C8), Leucine arylamidase, Valine arylamidase, Cystine arylamidase, Acid phosphatase, Naphthol-AS-BI-phosphohydrolase and alpha- Galactosidase | alpha- Chymotrypsin and alpha- Fucosidase |
| **API 50CH/E** |  |
| None | Glycerol, Erythritol, D-Arabinopyranose, L-Arabinose, D-Ribose, D-Xylose, L-Xylose, Ribitol, methyl β-D-xylopyranoside, D-Galactose, D-Glucose, D-Mannose, L-Sorbose, Galactitol, Inositol, Mannitol, Sorbitol, methyl α-D-mannoside, methyl α-D-glucopyranoside, N-Acetyl-D-glucosamine, Amygdalin, Salicin, Cellobiose, Lactose, Melibiose, alpha-Trehalose, Inulin, Melezitose, Raffinose, Glycogen, Erythritol, Gentiobiose, D-Xylose, D-Tagatose, L-Arabitol, D-Gluconic acid, 2-Keto-D-gluconic acid and 5-Dehydrogluconate |
| **BIOLOG**  α-D-Glucose, Glucuronamide | N-Acetyl Neuraminic Acid, 3-Methyl Glucose, D-Mannitol, D-Glucose-6-PO4, D-Aspartic Acid, D-Serine, Gelatin, L-Alanine, L-Histidine, L-Pyroglutamic Acid, Mucic Acid, p-Hydroxy-Phenylacetic Acid, Methyl Pyruvate, D-Lactic Acid Methyl Ester, Citric Acid, D-Malic Acid, L-Malic Acid, Bromo-Succinic Acid, Tween 40, α-Hydroxy-Butyric Acid, β-Hydroxy-D L-Butyric Acid |
| **Sensitivity assays** |  |
| Tetrazolium Violet, Tetrazolium Blue | 8% NaCl |

**Table S2:** Antibiotic Susceptibility Test (AST) Results for Four Strains Using G-XXII-minus minus (Catalog No. 0D061; Himedia, India) and Dodeca Universal-II Discs (Catalog No. DE007; Himedia, India). The table provides the results of AST assays performed on control strain as suggested by the manufacturer and UC10^T^. The antibiotics tested, their concentrations, zones of inhibition (in mm), and the inferred susceptibility (Highly Sensitive, Sensitive, Intermediate, or Resistant) are displayed.

| **Antibiotic (Symbol)** | **Conc.** | ***Staphylococcus aureus* (ATCC 25923)** | ***Escherichia coli* (ATCC 25922)** | ***Pseudomonas aeruginosa* (ATCC 27853)** | ***Dyadobacter aurulentus* (UC10**^T^**)** |
| --- | --- | --- | --- | --- | --- |
| **Kit: G-XXII-minus (Catalog No. 0D061; Himedia, India)** | | | | | |
| **Chloramphenicol (C)** | 30μg | 27 mm (Sensitive) | 23 mm (Sensitive) | Resistant* | 22 mm (Sensitive) |
| **Ampicillin (AMP)** | 10μg | 30 mm (Sensitive) | 19 mm (Sensitive) | Resistant | 18 mm (Intermediate) |
| **Tetracycline (TE)** | 30μg | 35 mm (Highly Sensitive) | 32 mm (Highly Sensitive) | 34 mm (Highly Sensitive) | 40 mm (Highly Sensitive) |
| **Gentamicin (GEN)** | 10μg | 23 mm (Sensitive) | 24 mm (Sensitive) | 29 mm (Sensitive) | 17 mm (Resistant) |
| **Streptomycin (S)** | 10μg | 28 mm (Sensitive) | 30 mm (Highly Sensitive) | 30 mm (Sensitive) | 10 mm (Resistant) |
| **Kanamycin (K)** | 30μg | 33 mm (Highly Sensitive) | 26 mm (Sensitive) | 10 mm (Resistant) | 28 mm (Sensitive) |
| **Co-Trimoxazole (COT)** | 25μg | 34 mm (Highly Sensitive) | 30 mm (Sensitive) | Resistant | 34 mm (Highly Sensitive) |
| **Amikacin (AK)** | 30μg | 22 mm (Sensitive) | 26 mm (Sensitive) | 24 mm (Sensitive) | 32 mm (Sensitive) |
| **Kit: Dodeca Universal-Il (Catalog No. DE007; Himedia, India)** | | | | | |
| **Colistin (CL)** | 10μg | 10 mm (Resistant) | 19 mm (Sensitive) | 17 mm (Sensitive) | 21 mm (Sensitive) |
| **Augmentin (AMC)** | 30μg | 32 mm (Sensitive) | 10 mm (Resistant) | Resistant | 16 mm (Intermediate) |
| **Netillin (NET)** | 30μg | 28 mm (Sensitive) | 20 mm (Sensitive) | 20 mm (Sensitive) | 16 mm (Intermediate) |
| **Norfloxacin (NX)** | 10μg | 34 mm (Highly Sensitive) | 40 mm (Highly Sensitive) | 37 mm (Highly Sensitive) | 40 mm (Highly Sensitive) |
| **Ceftriaxone (CTR)** | 10μg | 31 mm (Sensitive) | 35 mm (Highly Sensitive) | 24 mm (Sensitive) | 29 mm (Sensitive) |
| **Ciprofloxacin (CIP)** | 5μg | 40 mm (Highly Sensitive) | 40 mm (Highly Sensitive) | 31 mm (Sensitive) | 38 mm (Highly Sensitive) |
| **Cefotaxime (CTX)** | 30μg | 28 mm (Sensitive) | 36 mm (Highly Sensitive) | 23 mm (Sensitive) | 20 mm (Intermediate) |
| **Furazolidone (FR)** | 50μg | 21 mm (Sensitive) | 22 mm (Sensitive) | 23 mm (Sensitive) | 33 mm (Highly Sensitive) |
| **Amoxycillin (AMX)** | 10μg | 29 mm (Sensitive) | 13 mm (Resistant) | Resistant | 27 mm (Sensitive) |

*For organisms marked as 'Resistant,' the zone of inhibition was 0 mm, indicating no measurable inhibition around the antimicrobial disc.

**Table S3**. Cellular fatty acid content (%) of *Dyadobacter aurulentus* UC10^T^*,* and its closest phylogenetic neighbor *D. crusticola* and *D. luticola*. Values less than 1% are not mentioned.

| **Species** | ***D. aurulentus*** | ***D. linearis*** | ***D. crusticola*** | ***D. luticola*** |
| --- | --- | --- | --- | --- |
| **Type strain** | **UC10^T^** | **AB67^T^** | **CP183-8^T^** | **T17^T^** |
| **Acc. No.** | **MCC 4019** | **LMG 32342** | **DSM 16708** | **KCTC 52981** |
| **Saturated** |  |  |  |  |
| C_10:00_ |  | 1.7 |  |  |
| C_13:00_ | 4.1 | - | 2.4 | - |
| C_14:00_ | - | - | - | 1.9 |
| C_16:00_ | 28.9 | 14.6 | 21.7 | 18.7 |
| C_18:00_ | - | 7.8 | - | 2.2 |
| C_20:00_ | - | 1.2 | - | - |
| **Hydroxy** |  |  |  |  |
| C_13:0_ 2OH | 1.7 | - | - | - |
| C_15:0_ 3OH | - | 2.8 | - | - |
| C_16:0_ 3OH | - | 1.0 | - | 1.7 |
| C_17:0_ 3OH | - | 12.2 | - | - |
| **Branched** |  |  |  |  |
| C_12:0_ iso | 2.5 | - | 3.0 | - |
| C_13:1_ at 12-13 | - | - | - | 2.6 |
| C_14:0_ iso | 7.7 | - | 14.3 | - |
| C_15:0_ anteiso | 1.3 | 1.0 | - | - |
| C_15:0_ iso | - | 9.8 | - | 26.2 |
| C_15:0_ iso 3OH | - | 1.4 | - | 1.6 |
| C_15:1_ iso G | 4.9 | - | 2.9 | - |
| C_16:0_ iso 3OH | - | - | 1.3 | - |
| C_17:0_ iso | - | 1.4 | 2.1 | - |
| C_17:0_ iso 3OH | - | 4.3 | - | 3.3 |
| **Unsaturated** |  |  |  |  |
| C_16:1_ ω5c | 5.4 | 7.5 | 5.8 | 3.4 |
| C_17:1_ ω6c | - | 1.1 | - | - |
| C_18:1_ ω9c | - | 1.9 | - | - |
| Summed Feature 3 | 41.2 | 35 | 40.7 | 33.6 |
| Summed Feature 5 | - | 1.5 | - | - |
| Summed Feature 9 | - | - | 1.6 | - |
| Sum In Feature 3 | - | 25.8 | - | - |
| Sum In Feature 3* | - | 9.2 | - | - |
| Summed Feature 3 comprised C_16:1_ ω7c/C_16:1_ ω6c; Summed Feature 5 comprised C_18:2_ w6,9c/C_18:0_ ante and Summed Feature 9 comprised C_16:0_ 10-methyl/C_17:1_ iso ω9c. Sum In Feature 3 comprised of C_16:1_ w7c/C_16:1_ w6c while Sum In Feature 3* comprised of C_16:1_ w6c/C_16:1_ w7c | | | | |

**
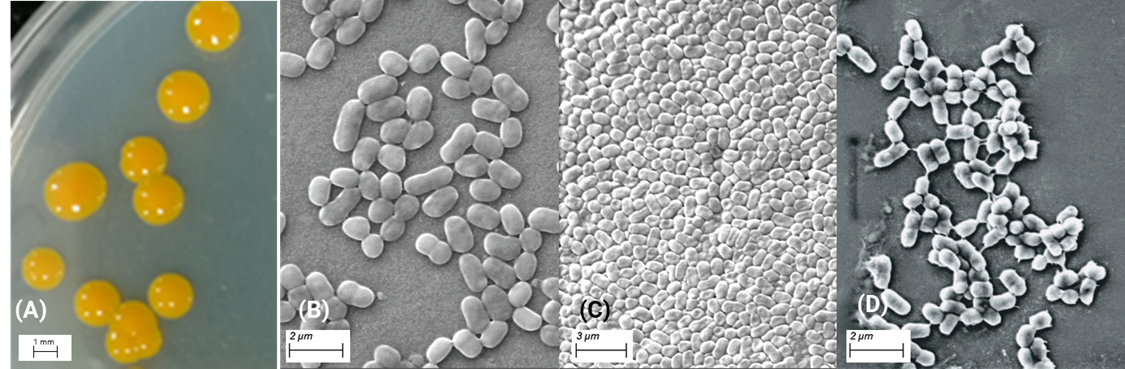
**

**Figure S1**. Colony and cellular morphology of *D. aurulentus* UC10ᵀ. Distinctly pigmented, convex, mucoid, golden colonies of UC10ᵀ grown on nutrient agar at 30°C for 6 days, highlighting its characteristic colony morphology (A); Scanning Electron Micrographs (SEM) of UC10^T^ illustrating its rod-shaped morphology (0.5–1.5 μm in length) (A). The strain aggregates predominantly in pairs, characteristic of the genus *Dyadobacter*; reflecting its name derived from "dyad," meaning a pair (C and D). Samples were sputter-coated with gold and imaged at 8500–13,500X magnifications using a Zeiss EVO18 microscope at 8 kV.

**
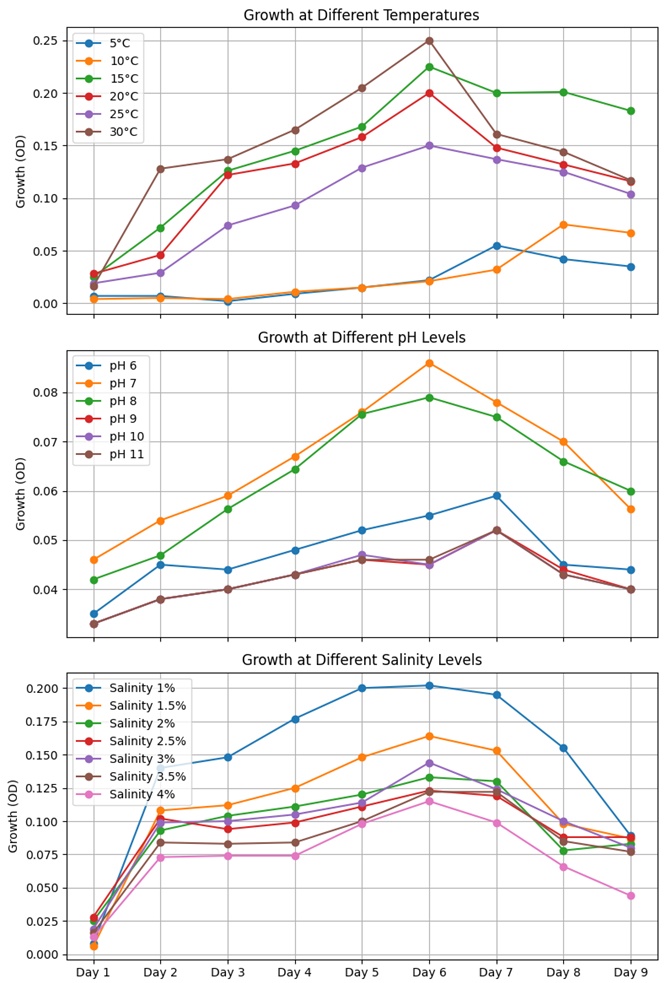
**

**Figure S2.** Growth of UC10^T^ under Different Environmental Conditions. The graphs illustrate the growth behaviour of UC10^T^ under different conditions, including temperature, pH, and salinity. Optimal growth was observed at 30°C, pH 7 and at 1% salinity.

**
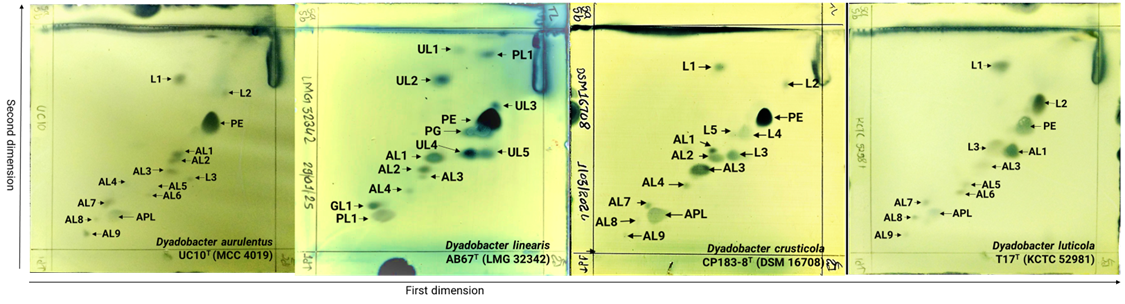
**

**Figure S3**. Polar lipid analysis was conducted on *D. aurulentus* UC10^T^ and its closest phylogenetic neighbors, *D. linearis*, *D. crusticola* and *D. luticola*. Total lipids were visualized using two-dimensional thin-layer chromatography (TLC) on silica gel, developed with appropriate solvent systems, and stained with 5% ethanolic phosphomolybdic acid. Phosphatidylethanolamine (PE) was the predominant lipid in all strains, with additional amino lipids (AL), aminophospholipids (APL), and unidentified polar lipids (UL) detected across the species. Notably, *D. linearis* exhibited phosphatidylglycerol (PG) and an unidentified glycolipid (GL1), absent in UC10ᵀ, while *D. luticola* was characterized by a dominant unknown polar lipid (L2). All data were derived from this study, illustrating species-specific variations in lipid composition.

**
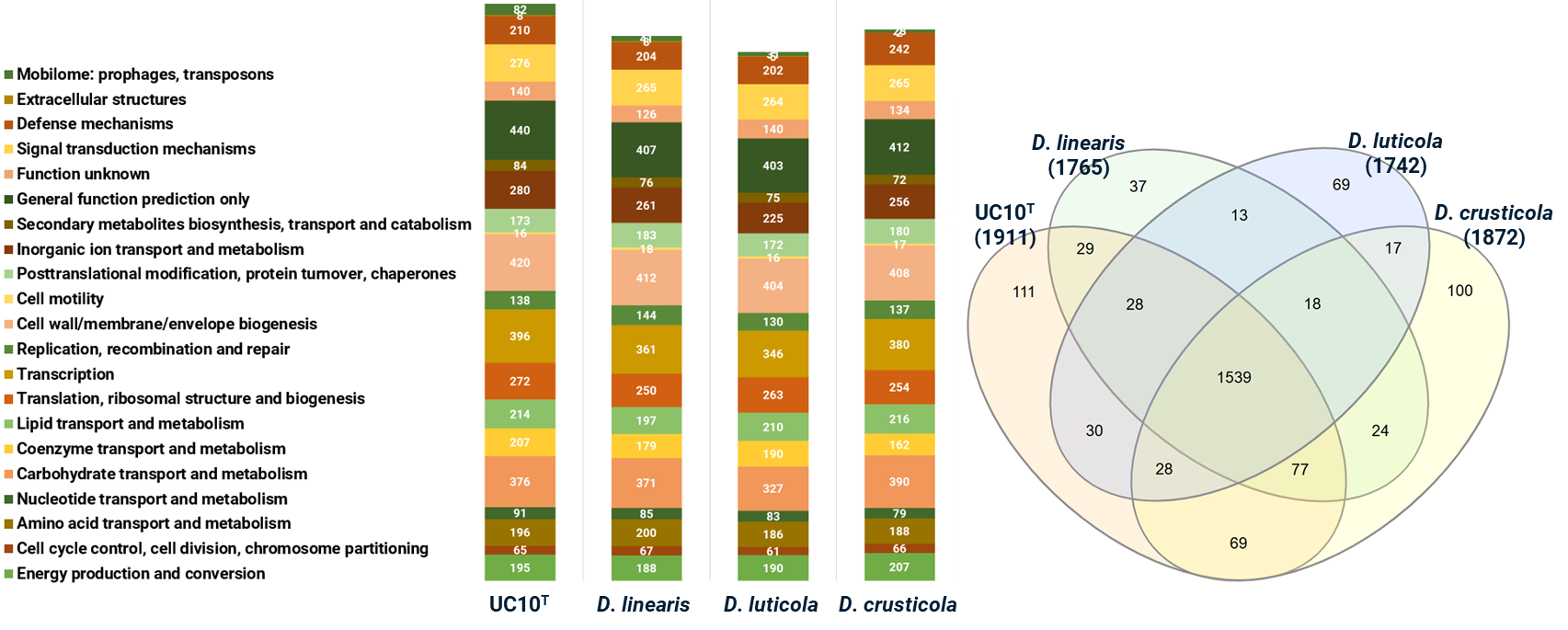
**

**Figure S4.** Comparative genome statistics illustrated through a Venn diagram (right) (39), highlighting shared and unique Clusters of Orthologous Groups (COGs) among *D. aurulentus* (VSRN01), *D. linearis* (CAJRAU01), *D. luticola* (VCEJ01), and *D. crusticola* (JNJB01). The distribution of COGs within shared genes is also depicted. COG functional categories (left) were assigned using DeepNOG (40), with predictions below a confidence threshold of 0.5 excluded. The results were cross-validated against NCBI’s COG database. In cases where genes were associated with multiple COG classes, the first-listed category was selected.


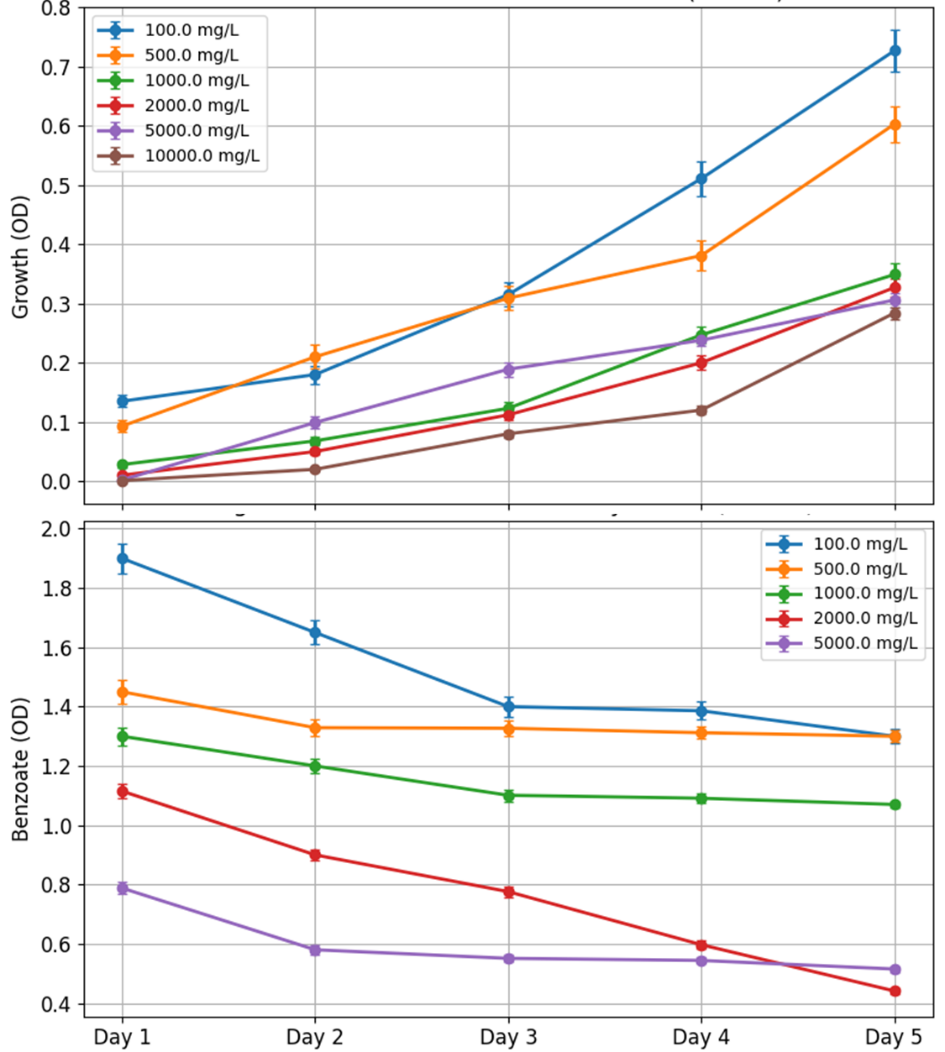


**Figure S5.** Growth and degradation assays of *D. aurulentus* UC10ᵀ in the presence of sodium benzoate. Growth of UC10ᵀ in nutrient broth supplemented with increasing concentrations of sodium benzoate (100 to 10,000 mg/L) measured as optical density at 600 nm over five days, indicating the strain’s ability to utilize sodium benzoate as a sole carbon and energy source (top). Degradation of sodium benzoate by UC10ᵀ in liquid mineral medium (LMM) measured at 230 nm over five days, demonstrating a concentration-dependent reduction in benzoate levels (bottom). Each value represents the mean of triplicate assays.
